# 3D Orthodontics - from Verne to Shaw

**DOI:** 10.1590/2176-9451.19.6.012-013.edt

**Published:** 2014

Imagination is the beginning of creation. You imagine what you desire, you will what you imagine and at last you create what you will. George Bernard Shaw

As children receiving new toys, we are approaching a great new world of fantasy. Three-dimensional image devices arrived at the port of Orthodontics with increasingly strong anchors firmly holding to the ground. In this case, immobility is symbolical and inversely proportional to the ongoing possibilities we are not even capable to imagine. Perhaps, the French novelist Julio Verner could.

Whereas technological development proves hasty, rendering 3D technology popular in Brazil walks at a low pace. High costs are the obstacle; however, as in any new technology, they will decrease when strong competition causes market spreading. With lower costs, orthodontists are able to use technology to a larger extent. Those who doze off will be left out of the market and miss the pleasure of envisioning and applying the splendor of human creativity.

The costs of CT scanners have decreased, although in a slow manner. Cost reduction has been mainly offered as a result of market competition, since no measures have been taken by the government in this regard. The taxes on imports hinder access to such tools which are indispensable for effective diagnosis and treatment. Conversely, accuracy of CT scans has significantly increased, thereby enabling microscopic diagnosis of pathologies such as ankylosis and root fracture.

In addition to computed tomography, other 3D tools also offer undeniable possibilities that allow clinicians to acquire 3D scans. In no time, photographs and dental casts as used nowadays will be replaced by 3D scanning of the human face and dentition. There is equipment available to this end; however, access is hampered by high costs. The aforementioned tools allow us to examine soft and hard tissues in an infinite variety of ways with resolution near microscopy. Tissue color is also reliably captured.

Orthodontics has been investigating the reliability of measurements obtained by means of several 3D tools. At first blush, the reliability of CT scans was successfully proved.^1^^,^ 2 Subsequently, images acquired by means of dental cast scanning proved perfectly feasible for clinical and scientific use.^3^^,^ 4 Scientific results also confirm the reliability of 3D face scans acquired with scanners or by means of stereophotogrammetry.^5^^,^ 6

Intraoral scanners have been recently tested, making us foresee the end of impressions and casts. A number of researches point to the excellent reproducibility of this method in comparison to conventional methods used to acquire dental casts.^7^^,^ 8 Nevertheless, when the time required to acquire and rendering scans is taken into account, the conventional method has proved more advantageous.^8^ Another disadvantage reported by patients is the greater discomfort provided by intraoral scanners.^8^ Their high costs remain a hurdle for the clinical practice. Despite the aforementioned drawbacks, it seems only a matter of time before we have faster and more comfortable 3D intraoral scanners. Market competition and technological development will certainly lead to cost reduction.

Imagination is endless. The images acquired might be reformatted into physical dental casts - similar in shape, volume and color - by means of 3D printers of which cost has significantly decreased. In no time plaster casts will be replaced by nylon or polymer ones. As a result, the patient will be provided with a physical copy of his dental arches or his face printed by a 3D printer; or they might even be able to receive digital files and print them at home using their own 3D printer.

At the pace of orthodontic appliance customization, the recent improvements in 3D printers and human creative capacity, I wonder whether we will be capable of, one day, designing and printing individual orthodontic brackets and archwires at our offices. Should that be the case, we will be able to customize angulation, tipping, size or even the material brackets are made of. Moreover, we might be able to perform bends and manufacture individual orthodontic archwires. Furthermore, this technology might allow us to, by means of a FEM model, foresee movement produced by archwires and brackets recently printed, even before we install the archwire. Orthodontists will be able to manufacture their own aligners and at each appointment predict the movement they produce. 

A new Orthodontics, based on immeasurably accurate diagnosis, is arising. Do you have any doubts? Shaw, the only man who won the greatest prizes - in science, the Nobel; and in arts, the Oscar - would not.

David Normando - Editor-in-chief (davidnormando@hotmail.com)
